# A new small molecule DHODH-inhibitor [KIO-100 (PP-001)] targeting activated T cells for intraocular treatment of uveitis — A phase I clinical trial

**DOI:** 10.3389/fmed.2022.1023224

**Published:** 2022-10-17

**Authors:** Stephan Thurau, Christoph M. E. Deuter, Arnd Heiligenhaus, Uwe Pleyer, Joachim Van Calster, Talin Barisani-Asenbauer, Franz Obermayr, Stefan Sperl, Romana Seda-Zehetner, Gerhild Wildner

**Affiliations:** ^1^Department of Ophthalmology, University Hospital, LMU München, München, Germany; ^2^University Eye Hospital, Eberhard-Karls-University, Tübingen, Germany; ^3^Department of Ophthalmology, St.-Franziskus-Hospital, Münster, Germany; ^4^Department of Ophthalmology, Charité — Universitätsmedizin Berlin, Corporate Member of Freie Universität Berlin and Humboldt-Universität zu Berlin, Berlin, Germany; ^5^Department of Ophthalmology, University Hospitals Leuven, Leuven, Belgium; ^6^Medical University of Vienna, Vienna, Austria; ^7^Panoptes Pharma GmbH, Vienna, now Kiora Pharmaceuticals Inc., Vienna, Austria; ^8^Epics Therapeutics, Gosselies, Belgium

**Keywords:** uveitis, humans, experimental autoimmune uveitis, rat, intravitreal therapy, clinical phase 1 trial, macular edema, visual acuity

## Abstract

Uveitis is a T cell-mediated, intraocular inflammatory disease and one of the main causes of blindness in industrialized countries. There is a high unmet need for new immunomodulatory, steroid-sparing therapies, since only ciclosporin A and a single TNF-α-blocker are approved for non-infectious uveitis. A new small molecule inhibitor of dihydroorotate dehydrogenase (DHODH), an enzyme pivotal for *de novo* synthesis of pyrimidines, has a high potency for suppressing T and B cells and has already proven highly effective for treating uveitis in experimental rat models. Systemic and intraocular application of KIO-100 (PP-001) (previously called PP-001, now KIO-100) could efficiently suppress rat uveitis in a preventive as well as therapeutic mode. Here we describe the outcome of the first clinical phase 1 trial comparing three different doses of a single intraocular injection of KIO-100 (PP-001) in patients with non-infectious posterior segment uveitis. No toxic side effects on intraocular tissues or other adverse events were observed, while intraocular inflammation decreased, and visual acuity significantly improved. Macular edema, a sight-threatening complication in uveitis, showed regression 2 weeks after intraocular KIO-100 (PP-001) injection in some patients, indicating that this novel small molecule has a high potential as a new intraocular therapy for uveitis.

**Clinical trial registration:**

[https://www.clinicaltrials.gov/ct2/show/NCT03634475], identifier [NCT03634475].

## Introduction

Within the group of intraocular inflammatory diseases, non-infectious uveitis is supposed to be of autoimmune origin. It often runs a chronic or relapsing course and has a high risk of visual deterioration and burden of illness, especially if the posterior segment of the eye is involved. Systemic treatment modalities include corticosteroids, disease-modifying anti-rheumatic drugs, ciclosporin A and adalimumab, a TNF-alpha inhibitor (1, 2). Intravitreal treatments consist of corticosteroids only, which are associated with significant local side effects like cataract and glaucoma (3). Therefore, novel intraocular anti-inflammatory strategies are still an unmet need.

The disease is mediated by T helper cells of the Th1 and Th17 type, which must be activated to be able to pass the blood eye-barriers that protect the immune privileged eye from assaults of the immune system (4–6). Activated leukocytes may enter the inner eye and screen for potential pathogens, but only those T cells that recognize their antigen presented within the eye can get reactivated to secrete chemokines and cytokines for recruiting inflammatory cells like macrophages and granulocytes to the eye, which cause the typical signs of inflammation and tissue destruction (7, 8).

Upon activation, T cells but also B cells have an increased need of nucleotides for mRNA and DNA synthesis. Activated T cells have an 8-fold increased need of pyrimidines, which cannot be provided from the salvage pool, therefore *de novo* synthesis of nucleobases must be initiated (9–11). A pivotal enzyme for the synthesis of pyrimidines is dihydroorotate dehydrogenase (DHODH), which can be blocked by the small molecule KIO-100 (PP-001) 150-times more potently than by leflunomide (12–14).

To prove the efficiency of KIO-100 (PP-001) on uveitis we have used two rat models of experimental autoimmune uveitis (EAU), a chronic, clinically monophasic disease with chorioretinal neovascularization as later sequel, and a spontaneous relapsing-remitting disease. In both models we first applied the small molecule orally to obtain a systemic immunosuppression for preventing uveitis by treatment starting with immunization, but we were also successful with late treatment in ongoing disease, reducing relapses and neovascularization. *In vitro*, rat T cell proliferation and cytokine secretion was significantly suppressed in a dose-dependent manner (13).

To avoid a general systemic immunosuppression, we also applied the small molecule locally by intraocular injection into rat eyes (15). The rationale of this experiment was to inhibit the intraocular reactivation of the autoreactive T cells and their ability to recruit inflammatory cells. We could show that a single intraocular injection of KIO-100 (PP-001) after the resolution of the primary course of relapsing EAU significantly inhibited the frequency and intensity of relapses in the small molecule-treated eyes compared with vehicle treatment. According to the pharmacokinetics (PK) performed in rabbits the concentration of the injected KIO-100 (PP-001) in the eye had dropped to 10% after 12 h and was not detectable any more after 96 h, while the immunosuppressive effect in the rat model was lasting for at least 6 days. We hypothesized that the intravitreally injected small molecule targeted the T cells, which were present in the eye at that time and prevented their reactivation and thus the induction of recurrent inflammation. No adverse effects on intraocular tissues were observed, and *in vitro* investigations with a human retinal pigment epithelial cell line revealed no toxic effect on this cell type with respect of viability, proliferation, and cytokine/chemokine production (15).

Here we show the results of a prospective, first in man phase 1 clinical trial in patients with posterior uveitis, the type of autoimmune disease, which has the greatest risk for retinal destruction and permanent vision loss. Twelve patients with chronic, non-infectious, bilateral uveitis from six centers received a single intravitreal injection of 0.3, 0.6, or 1.2 μg KIO-100 (PP-001), respectively. In addition to the assessment of general and uveitis-specific ocular symptoms and visual acuity, pharmacokinetic evaluation of plasma levels of the study drug was performed. Except for some minor side effects related to the injection procedure but not to the study drug we found no major systemic or ocular side effects, especially no increase of intraocular pressure. Plasma levels of the small molecule were below the detection limit in our patients. Within the first week visual acuity increased in a dose-dependent fashion in all eyes until the end of the study on day 28, despite a very short intraocular half-life of the study drug. In addition to a regression of intraocular inflammation a decrease of retinal thickness was found in 3 of 4 eyes 2 weeks after receiving the 1.2 μg dose and a reduction of cystoid macular edema (CME) in two eyes with doses of 0.6 or 1.2 μg. Thus, in this phase 1 trial the new DHODH inhibitor KIO-100 (PP-001) presented as a potential and promising new drug for the intraocular treatment of uveitis.

## Materials and methods

### Study drug

The study drug KIO-100 (PP-001) is a new small molecule inhibitor of DHODH, a mitochondrial enzyme that is required for the *de novo* synthesis of pyrimidine nucleotides, and thus impeding DNA and RNA synthesis.

**FIGURE 1:**
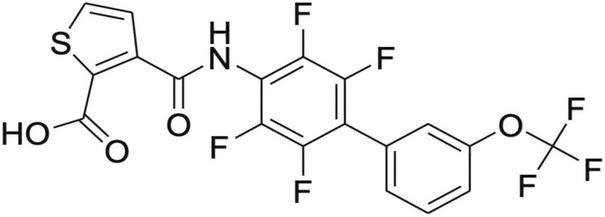
Structure of KIO-100 (PP-001). See also compound 13 in Leban et al. (16).

KIO-100 (PP-001) is a biphenyl-4-ylcarbomyl thiophene carboxylic acid derivative (Figure 1) with a molecular mass of 479,3 D and has a 50% inhibitory concentration for DHODH of less than 4 nM and therefore a 150-fold higher potency than leflunomide (IC50 of 650 nM), an established drug with a similar mode of action (16). In addition, KIO-100 (PP-001) is highly specific for DHODH and does not inhibit tyrosine kinase or cause hepatotoxicity like leflunomide since, like other small molecule DHODH inhibitors, it does not need to be converted from a prodrug to its active form in the liver (16–18).

Neither the preclinical uveitis rat models nor toxicology studies in rabbits have shown any toxic effects of the small molecule on intraocular cells/tissue at relevant exposure (15). Pharmacological investigations revealed that the amount of the study drug in the plasma of rabbits after intravitreal injection of high doses of KIO-100 (PP-001) was negligibly low. The half-life of KIO-100 (PP-001) in rabbit eyes after intravitreal injection was between 12 and 21 h, and all toxicity studies proved safety of this drug. The study drug was applied once intravitreally in a sterile, buffered, isotonic aqueous solution.

### Patient selection

The study protocol and the informed consent form were reviewed and approved by the relevant Independent Ethics Committees (IEC) at each center prior to the start of the study (see Supplementary Table 1). Patients had to give their written informed consent. The study was registered with the Paul-Ehrlich-Institute in Langen, Germany and listed at ClinicalTrials.gov Identifier: NCT03634475.

For this first-in-man, open-label, phase-1 clinical trial 12 patients with bilateral chronic, non-infectious intermediate, posterior or pan-uveitis as defined by the Standardization of Uveitis Nomenclature (SUN) Working Group (19) were selected to receive a single intravitreal injection of KIO-100 (PP-001) in one eye. All patients had structural and vision threatening complications. For each patient only the worse eye was injected. Male or female patients at the age of at least 18 years were eligible and had to use two combined methods of contraception if females or female partners of male participants were in an age of childbearing potential. All females had to undergo a negative pregnancy test. Systemic or ocular infection had to be ruled out. Best corrected ETDRS (Early Treatment Diabetic Retinopathy Study) visual acuity had to be better than 10 (20/630), and worse than 50 letters (20/100) in the study eye, and 70 letters (20/40) or better in the fellow eye.

Any disease activity was acceptable, so there was no minimum requirement for disease activity according to the SUN criteria (19), since treatment effects as frequently referred to in phase 2 or 3 clinical trials were not a primary target in this phase 1 trial.

Most importantly, exclusion criteria included significant media opacities, any ongoing systemic treatment with biologicals or cytostatic agents, fluocinolone implants within the past 3 years, dexamethasone-implants within 6 months or any other intravitreal injection within 90 days before injection with KIO-100 (PP-001) (day 0). Patients with uncontrolled glaucoma, hypotony, aphacic eyes or eyes with anterior but not posterior chamber lens were also excluded; see Supplementary Table 2 for the complete list of inclusion and exclusion criteria.

The primary study objective was to assess the safety and tolerability of ascending doses of KIO-100 (PP-001) in patients with chronic, non-infectious uveitis when administered as a single intravitreal injection of 100 μl containing 0.3, 0.6, or 1.2 μg KIO-100 (PP-001) (*n* = 4/group). Secondary objectives included the assessment of improvement of inflammation, and the pharmacokinetics (PK) of KIO-100 (PP-001) in plasma at screening, 4 ± 1 h after intravitreal injection and on day 2.

Patients were examined at screening, baseline, pre and post injection days 2, 7, 14, and 21 and at the final exit visit on day 28 after injection. A safety telephone call was scheduled on day 1.

Assessments and procedures are listed in Table 1.

**TABLE 1:** List of assessments and procedures.

Best corrected visual acuity (ETDRS)
Slit lamp examination
Intraocular pressure
Dilated fundoscopy
Corneal endothelial microscopy
Optical coherence tomography
Fluorescein angiogram
Amsler grid
Fundus photography
Visual field (computerized 30°)
Electroretinography
Visually evoked cortical potential
Study drug injection
Blood sampling for PK analysis
Urine pregnancy test
Medical and ophthalmic histories
Vital signs
Twelve-lead electrocardiogram
Laboratory assessments
Patient-reported outcomes
Concomitant medication
Serious medical events
Adverse events

For safety reasons the minimum time interval between injection of patients was 7 days to ensure sufficient time for development of adverse events before injecting the next patient. After each group of four patients and before proceeding to the next higher dose of KIO-100 (PP-001) a Data Safety Monitoring Board evaluated the safety of the previous dosing group after the final visit (day 28) of the last patient and had to consent on proceeding to the next higher dosing group.

### Surgical procedure

Injection of the study drug was performed in an operating room or surgical suite using sterile technique. The final diluted KIO-100 (PP-001) solution had to be clear and colorless and was injected at the required dose in a volume of 100 μl. For safety reasons it was mandatory for patients to remain in the hospital for at least 4 h after the injection procedure.

## Results

### Demographic of patients

Twelve patients (eight females and four males) with an average age of 52 years (range 27–69) were included. All patients completed the study until the final visit. Average duration of uveitis was 10 years (range 13 months to 23 years). At baseline average visual acuity was log[MAR] (logarithm of minimum angle of resolution) 0.87 (range 1.3–0.4), which is equivalent to 20/150 (range 20/400–20/50). For assignment of diagnosis to dosing groups see Table 2.

**TABLE 2:** List of diagnosis and group assignment.

Group	List of diagnosis
0.3 mg	Panuveitis (*n* = 1), intermediate uveitis (*n* = 1), and idiopathic posterior uveitis (*n* = 2)
0.6 μg	Serpiginous choroiditis (*n* = 1), choroiditis (*n* = 1), and intermediate uveitis (*n* = 2)
1.2 μg	Serpiginous choroiditis (*n* = 1), panuveitis (*n* = 1), intermediate uveitis (*n* = 1), and idiopathic posterior uveitis (*n* = 1)

### Safety measurements

Systemic and ocular safety as well as PK analysis from peripheral blood were investigated.

### Systemic safety

Vital signs were recorded at each visit in the study center. No substantial changes from screening to Day 28 in mean values of all blood chemistry parameters (blood urea nitrogen, creatinine, glucose, sodium, potassium, chloride, calcium, magnesium, phosphorus, aspartate aminotransferase (AST), alanine transaminase (ALT), gamma-glutamyltransferase (gamma-GT), alkaline phosphatase, creatine phosphokinase, total and direct bilirubin, uric acid, albumin, and total protein) were observed in all treatment dose cohorts. Minor changes were seen in the 0.3 μg group for creatinine, potassium, phosphorus, creatine phosphokinase (CPK), gamma-GT and albumin, in the 0.6 μg group for CPK and in the 1.2 μg group for CPK, gamma-GT and alkaline phosphatase. None of these changes were categorized as clinically significant. Twelve-lead electrocardiogram at base line and final visit on day 28 did not show any pathologic changes in any patient. Patients also did not report about any health issue potentially associated with the study, and no serious medical events nor systemic adverse events were observed.

### Blood sampling for pharmacokinetics analysis

For PK analysis blood was drawn at screening, 4 h after intravitreal injection, and on day 2. KIO-100 (PP-001) was not reported in any of the samples. The quantification limit of the assay was 10 ng/ml.

### Ocular findings

#### Procedure related events

Injection-related events occurring with the procedure included a transient increase in intraocular pressure in one eye, and mild conjunctival hemorrhages or conjunctival hyperemia at the injection site in all eyes. No intravitreal complications were observed. None of these complications were considered to be substance related.

#### Visual acuity

**FIGURE 2:**
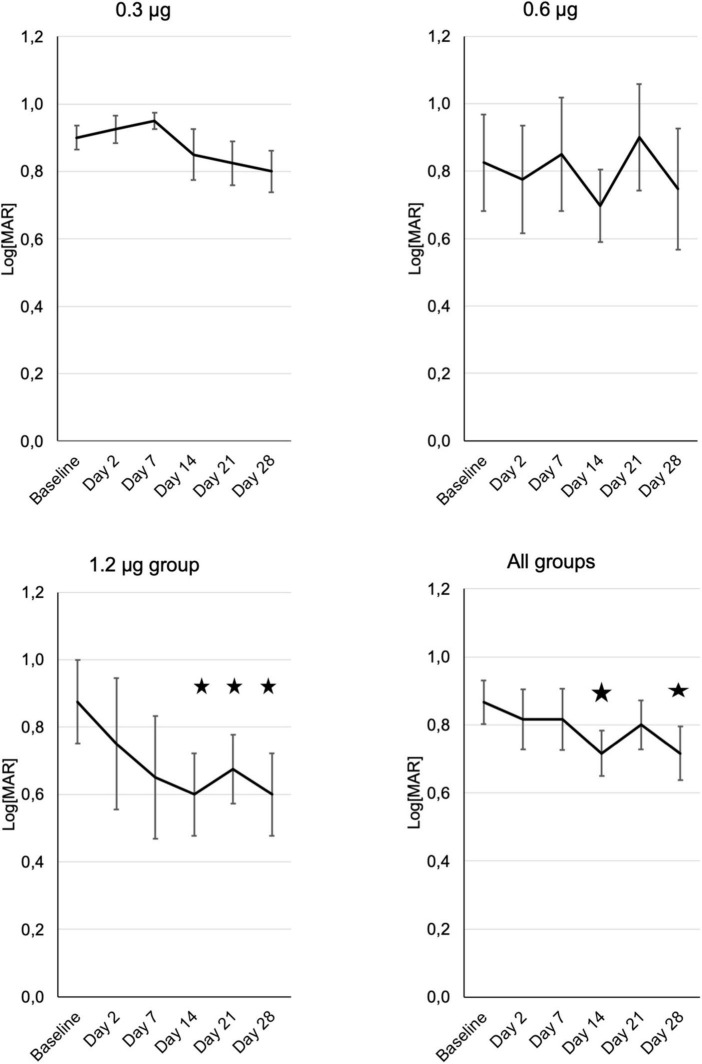
Development of visual acuity in the three treatment groups (*n* = 4) from baseline until the end of the study at day 28, assessed at the respective visits. “All patients” shows the means of visual acuity ± SE from all patients of the different treatment groups. Note that decreasing Log[MAR] values represent increasing visual acuity. Asterisks represent significant (*p* ≤ 0.05) differences to baseline.

Visual acuity was tested using ETDRS charts and results were converted to log[MAR]. On the log[MAR] scale smaller numbers represent better visual acuity. In the PP-001/0.3 μg group visual acuity increased slightly throughout the study. Patients receiving 0.6 μg had an improved VA at day 14 by one line, and in the 1.2 μg-group VA improved by 0.3 log[MAR], which represents an increase by three lines or a doubling in resolution, at days 14 and 28. In the high-dose group VA improved already on day 2, continued to increase until day 14 and remained stable until day 28. This increase was statistically significant (*p* ≤ 0.05) in this group at day 14, 21, and 28. None of the eyes had a loss of VA of more than two lines compared to baseline at any time point (Figure 2).

#### Intraocular pressure

**FIGURE 3:**
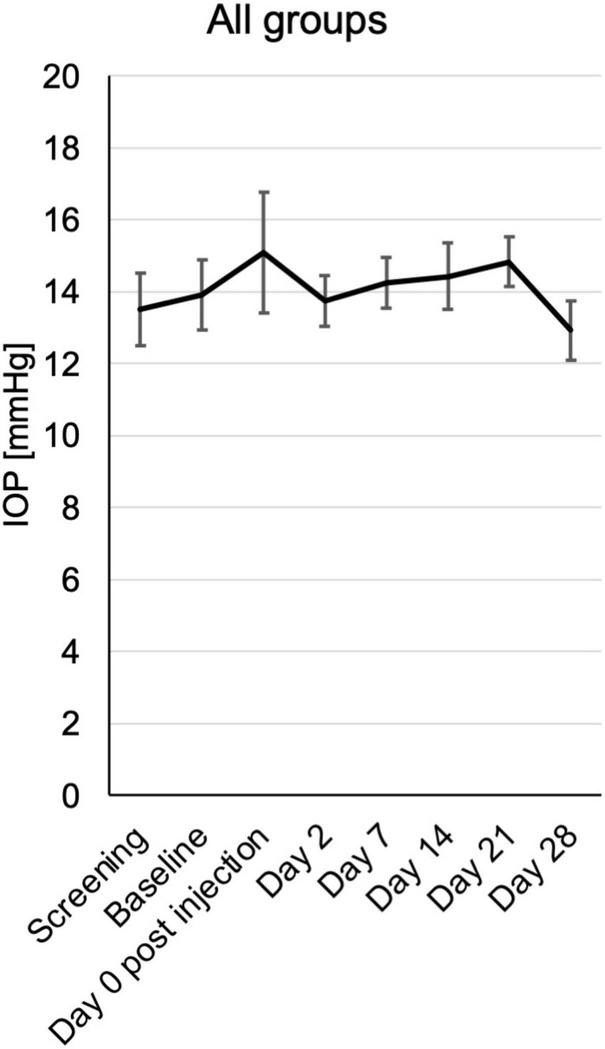
Intraocular pressure (IOP) from baseline until the end of the study at day 28, assessed at the respective visits. Mean mmHg ± SE is shown for all patients from the different treatment groups.

In a single case a transient increase of intraocular pressure (IOP) to 32 mmHg was observed directly after the injection. In all other cases and time points the IOP remained within the normal limits between 7 and 19 mmHg. There was also no trend toward an increase or reduction within the follow up in any group (Figure 3 and Supplementary Figure 1).

#### Inflammatory cells in the anterior chamber and vitreal haze

Signs of active inflammation according to the SUN were very subtle or negative in most patients at inclusion. According to SUN grading scheme, six ordinal grading steps are defined (0+, 0.5+, 1+, 2+, 3+, and 4+) for anterior chamber (AC) cells as well as the vitreal haze (19, 20).

**FIGURE 4:**
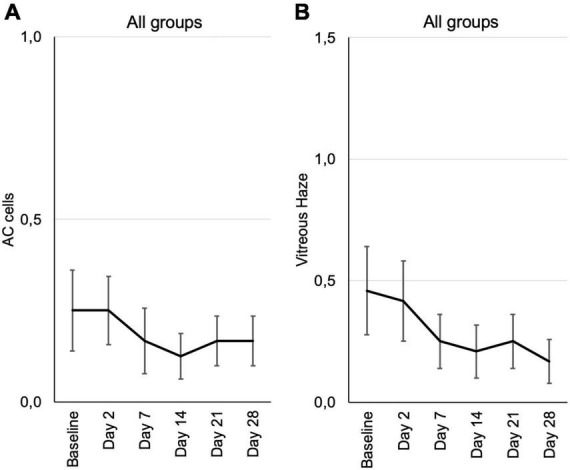
Inflammation parameters according to the SUN grading from baseline until the end of the study at day 28, assessed at the respective visits. **(A)** Cells in the anterior chamber (AC cells). **(B)** Vitreous haze. Mean grading score of AC cells ± SE **(A)** and mean vitreous haze scores ± SE **(B)** are shown for all patients from the three different dosing groups at each visit. According to the SUN grading scheme for AC cells and vitreous haze six grading steps are defined (0+, 0.5+, 1+, 2+, 3+, and 4+) (19, 20).

There were only two patients with 1+ anterior chamber cells in this study, who were randomly assigned to the 0.6 μg KIO-100 (PP-001) group. In both patients AC cells as well as vitreal haze improved within 7 days after injection. All other patients had 0.5+ or less cells and therefore a therapeutic effect on inflammation could not be detected (Figure 4A and Supplementary Figure 2).

Vitreal haze in the 0.3 μg group had increased on day 2 and showed a trend toward improvement in the follow up, also in the 0.6 μg group with an improvement by one step on the SUN scale. In the group receiving 1.2 μg KIO-100 (PP-001) only one patient had trace (0.5+) haze at baseline, which had disappeared after 2 weeks (Figure 4B and Supplementary Figure 3).

Since this phase 1 trial was aiming at safety of the drug, disease activity was not an inclusion criterion and therefore only a trend of a positive treatment effect could be determined. However, no worsening but rather an improvement has been observed.

### Central retinal thickness

**FIGURE 5:**
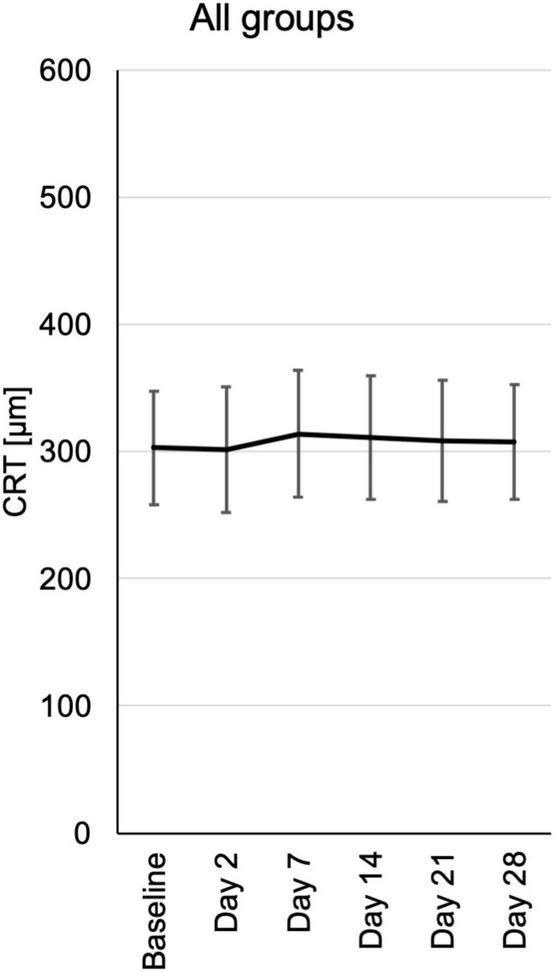
Central retinal thickness (CRT) from baseline until the end of the study at day 28, assessed at the respective visits. CRT measurements were extracted from automated standard OCT (optical coherence tomography) of the central fovea of the macula. CRT is depicted for each visit of all patients pooled from the three different dosing groups.

Central retinal thickness (CRT) was measured by optical coherence tomography (OCT). During the follow-up there was no significant change in CRT. Nevertheless, there was a trend toward reduced CRT in the 0.6 and 1.2 μg groups at day 2 and days 2, 7, and 14, respectively (Figure 5 and Supplementary Figure 4).

**FIGURE 6:**
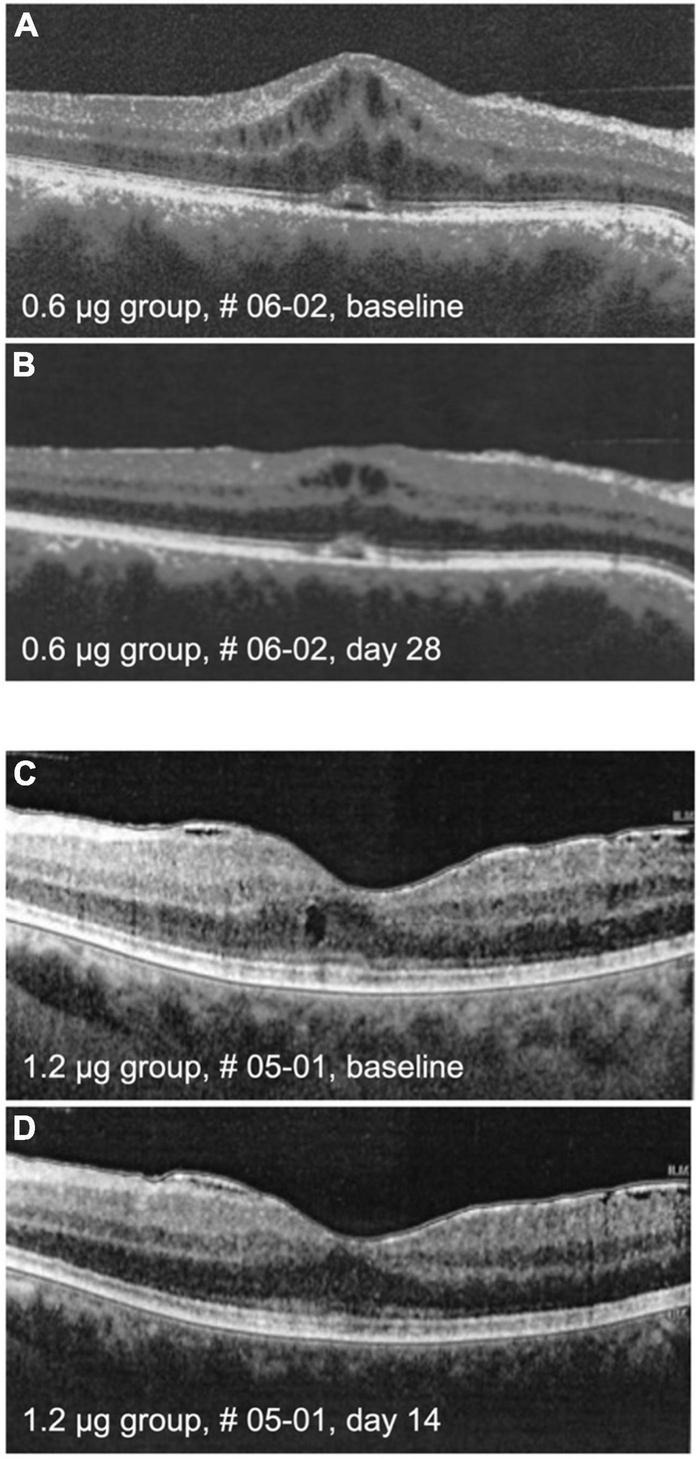
Optical coherence tomography (OCT) images. The upper panel shows macula of patient # 06-02 at baseline **(A)** and on day 28 **(B)** after intraocular injection of 0.6 μg KIO-100 (PP-001). Note a clinically meaningful reduction of cystoid macular edema (CME) in the region of the macula. In the lower panel the macula of patient # 05-01 is shown at baseline **(C)** and at day 14 **(D)** after injection of 1.2 μg of KIO-100 (PP-001). In this case the initial size of CME spaces was much smaller, but after injection the CME has disappeared completely.

Cystoid macular edema (CME) is an important feature of inflammatory macular disease and has been recorded separately from central macular thickness in this study. In the groups receiving 0.6 and 1.2 μg a total of five eyes had CME at the baseline examination which significantly improved clinically in two eyes, one in the 0.6 and one in the 1.2 μg-group (Figure 6). In these cases, the improvement of CME correlated with reduced CRT.

#### Electrophysiology

Electrophysiological testing included visually evoked cortical potentials (VECP) and electroretinography (ERG) and was performed according to the International Society for Clinical Electrophysiology of Vision (ISCEV)-standards (21) at screening or baseline and final visit at day 28. ERG was repeated at day 14 (visit 7) but was not available at all centers. Due to the destructive nature of the disease and the advanced stages of uveitis in this study most participants had pathological VECP and ERG findings at beginning, but during follow up there was no trend toward further elongation of latency or reduction of amplitude in VECP nor reduction of amplitudes in scotopic or photopic ERG.

#### Visual field

**FIGURE 7:**
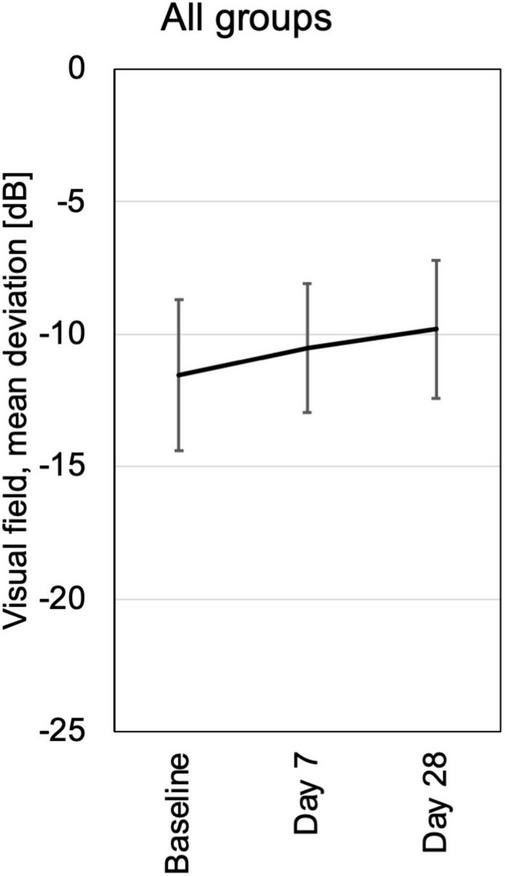
Visual field testing was performed at baseline, days 7 and 28. Mean deviation from normal is shown for each visit of all patients pooled from the three different dosing groups. All patients had loss of visual field sensitivity at baseline due to pre-existing destruction of previous chronic uveitis. In the follow up were no additional losses of more the 2 dB in a single patient. At day 28 the average visual field sensitivity had increased slightly, probably in part by training effects.

Computerized 30° visual fields at baseline, days 14 and 28 revealed a small but not significant trend toward improvement by a reduction of mean defects in all treatment groups. There were no losses of 2 dB (decibel) or more compared to baseline or any previous test in the follow up (Figure 7 and Supplementary Figure 5).

#### Fluorescein angiography

Standard fluorescein angiography (FA) was performed at screening and final visit (day 28). At screening FA showed the typical signs of uveitis with vascular leakage, retinal and papillary staining and hyperfluorescence, only one eye had a normal FA. In four eyes there was a reduction in retinal staining intensity (leakage) seen in all three dosing groups, while in the other eight eyes there was no change in fluorescein pathology.

#### Corneal endothelial microscopy

Corneal endothelial cells were counted at baseline, days 7 and 28 and remained stable throughout the study in all three dosing groups (Supplementary Figure 6).

## Discussion

Prior to the initiation of this phase 1 trial the effect of the small molecule DHODH-inhibitor KIO-100 (PP-001) had been demonstrated on rat EAU by preventive systemic (oral) application and revealed a dose-dependent, almost complete suppression of EAU. Using a spontaneous relapsing model of rat EAU a significant suppression of relapses was still obtained when the oral KIO-100 (PP-001)-treatment was initiated in a therapeutic mode after the resolution of the first course of uveitis (13). In a chronic model of EAU, where the autoreactive T cells secrete VEGF and thus induce chorioretinal neovascularization (CNV), also CNV-induction was significantly reduced by inhibiting T cells and their VEGF secretion when the oral KIO-100 (PP-001)-treatment was commenced during ongoing disease. Both, proliferation of rat and human T cells and their cytokine and chemokine secretion was suppressed by KIO-100 (PP-001) *in vitro* (13, 15). Cytokine production of RPE cells, shown with primary human RPE cells and the human ARPE-19 cell line, was not affected by treatment with KIO-100 (PP-001) (15).

The therapeutic potency of intraocular KIO-100 (PP-001) on uveitis was previously investigated in a spontaneously relapsing rat model of EAU (15). Therapeutic injection of KIO-100 (PP-001) was performed in diseased rat eyes after the first course of intraocular inflammation to prevent further relapses. Although the retinal concentration of KIO-100 (PP-001) rapidly and massively decreased within the first 8 h to about 30% of the injected dose, further dropped to about 5% after 12 h and was below the detection limit after 4 days, we observed a significant suppression of the frequency of relapses and their intensity within the first 6 days after injection (15). We hypothesized that the activation of intraocular T cells present in the eye at the time of the intravitreal injection was hampered by KIO-100 (PP-001) and T cells were therefore unable to recruit inflammatory cells to induce a relapse.

In this prospective, open labeled, controlled clinical trial the substance KIO-100 (PP-001) was administered to humans for the first time. The route of administration was once intravitreal with 3 different doses of 0.3, 0.6, and 1.2 μg. Side effects, deemed by investigators as not clinically meaningful, occurred with the surgical procedure including a single incidence of transiently increased intraocular pressure and conjunctival bleedings or conjunctival hyperemia.

The primary goal of this study was to demonstrate safety and tolerability of intravitreal injections of KIO-100 (PP-001). Basically, all measurements and clinical observations served this aspect. In none of the cases we observed any drug-related ocular side effect. Since the amount of the injected study drug was very low the systemic exposure in the peripheral blood was below the quantification level of the assay. We could neither detect any systemic nor ocular toxicity. In summary, all safety measures demonstrated KIO-100 (PP-001) to be safe at the tested doses within the observation period of 4 weeks.

Although the study design and patient selection were targeted at detecting ocular or systemic side effects and not at anti-inflammatory, therapeutic effects, we could observe a clinical improvement in the two groups receiving the higher doses, in which visual acuity increased 14 days after injection. This improvement was significant and still observed after 28 days in the highest dose group. Also, classical signs of inflammatory activity like anterior chamber cells and vitreal haze were reduced in the two higher dose groups demonstrating a potential positive effect on uveitis. However, since this study aimed at assessing safety of the study drug the patients assigned to the different dose groups differed with respect of diagnoses and disease intensities, which might have influenced the outcome of the three treatment groups.

The frequency of the relapses in rat EAU is much higher than in human uveitis, which might explain that the latency for recurrences of 6 days observed in rats was extended to at least 4 weeks without recurrent inflammation in patients. The observation period of this study ended 28 days after injection, therefore no data are available beyond this point. All rat eyes were investigated histologically after the termination of these experiments, and no alterations except for the usual signs of inflammation-induced retinal destruction from uveitis had been observed in both, the KIO-100 (PP-001) and the vehicle-treated groups.

Intraocular application of therapeutic drugs can avoid potential systemic side effects and will only affect the autoreactive cells in the target organ of the disease. The only presently available intraocular therapeutics for uveitis are corticosteroids, which can be introduced with sustained-release carriers and therapeutic effects between 4 months and some years. However, corticosteroids can have side effects in the eye, including cataract development and glaucoma (22). Due to the single application and the short half-life of KIO-100 (PP-001) we could not observe such adverse events within the 4 weeks observation time, especially no elevated intraocular pressure pointing to glaucoma development.

The effect of intraocular KIO-100 (PP-001) on signs of inflammation like cells and haze in the vitreous was visible only after 7–14 days, which points to suppressed T cell activation rather than to an immediate anti-inflammatory effect. This is in coincidence with previous *in vitro* data of peripheral blood lymphocytes from human donors showing that KIO-100 (PP-001) suppresses proliferation of lymphocytes and their cytokine secretion, but has no effect on the secretion of monokines, which suggests that monocytes/macrophages are not affected by DHODH inhibition (15).

Also, in the groups receiving higher doses, in 2 of 5 patients their pre-existing CME resolved transiently, associated with a decrease in central retinal thickness. Although in one patient the CME had previously been therapy-refractive and had not responded to systemic or intravitreal steroids including dexamethasone slow releasing devices, we could observe a regression of the edema after injection of KIO-100 (PP-001). Macular edema as a complication of uveitis accounts for about 40% of cases with visual impairment (23). Mast cells have been found in the uveal tract and are described to play a role in macular edema fostering leakage of blood vessels (24–30). DHODH-inhibition was shown to induce apoptosis in mast cells (31), which might explain the positive effect of KIO-100 (PP-001) on increased retinal thickness and macular edema, respectively, in addition to the suppression of T cells that fuel the inflammation by the activation of innate immune cells.

The T cell type responsible for the induction and maintenance of uveitis is not yet completely clear. In experimental mouse models an important role of Th1 and Th17 type cells for the pathogenicity was postulated (6, 32), which has led to the initiation of clinical trials targeting IL-17. Those trials had not been successful so far (33), explained by the fact that T helper cells and especially Th17 cells are not restricted to the production of one cytokine, but are producing a cocktail of different cytokines which are tuning their function (5, 34).

In our rat models of experimental autoimmune uveitis (EAU) we have shown that both, Th1 and Th17 cells, are differently expresses in diseased eyes depending on the type of uveitis. In a spontaneous relapsing-remitting rat model induced by immunization with R14, a peptide from interphotoreceptor retinoid-binding protein IRBP and adjuvant Th17 cells are obviously necessary to guide autoreactive T cells to the eye, since they are mainly found in the eyes at onset of the first attack of uveitis, which is suggesting a function in facilitating invasion of inflammatory cells. In the later course of EAU and especially during relapses Th1 cells are dominating (5, 35). The role of Th1 cells and their lead cytokine IFN-γ was further demonstrated by the induction of synchronized relapses after intraocular injection of IFN-γ. The other model, induced with retinal S-antigen peptide PDSAg, displays only one clinically visible inflammatory attack, which is then followed by chorioretinal neovascularization as a late sequel. This is caused by the VEGF secretion of the autoreactive, PDSAg-specific T cells, a rarely described feature of T lymphocytes (13, 36). This type of uveitis is initiated by an early intraocular invasion of predominantly Th1 cells, while Th17 cells are increasing at the resolution of inflammation, probably playing an important role as regulatory cells, since they are co-expressing IL-10. In our rat models we could demonstrate that the T cell populations within the eyes during uveitis are highly dynamic, they change their phenotype by co-expressing IFN-γ and IL-17, even together with IL10, indicating that they might convert to Tregs, since the IFN-γ/IL-17/IL-10 co-expressing cells increase at the resolution of the monophasic disease and decrease during the course of the relapsing uveitis. In late remission of the monophasic EAU the Foxp3+ Treg population is higher than in the eyes of rats with relapsing-remitting disease. This shift of T cell populations is only observed in the eyes, but not in the lymph nodes of the same animals (5). In human non-infectious uveitis, where only peripheral blood is available for investigation, Th17 cells were rather found to facilitate ocular invasion of inflammatory cells and later converting to T regs than driving the autoimmune response (37–39).

These points to the fact that within the tissue affected by the autoimmune disease T cell populations undergo changes of phenotype and potentially of their function that is not reflected by peripheral lymphocyte populations. That is, why local targeting of autoreactive T cells, here by injecting therapeutic drugs into the eyes, might have a different effect on the T cells and thus on the disease than just systemic treatment, suppressing the activity of the tissue-invading lymphocytes (5). When rats are concomitantly immunized with both antigens, the monophasic disease phenotype dominates, and the intraocular IL-10+ and Foxp3+ T cell populations correspond to the populations detected in the monophasic, PDSAg-induced disease, irrespective of whether the animals were immunized with a mixture or both antigens separately in contralateral legs (40). Interestingly, in both rat models, irrespective of a relapsing or monophasic course, green fluorescent protein (GFP) + autoreactive T cells can still be detected within the retinal tissue even weeks after resolution of intraocular inflammation (40). Those cells form clusters during spontaneous relapses, suggesting intraretinal expansion of the remaining autoreactive T cells. They might be a reservoir for recurrences or fuel subclinical inflammation, and moreover, they seem to attract newly invading T cells (40). Those cells would be targeted by intraocular, T cell suppressing treatment as proposed and tested here in this clinical phase 1 trial.

The role of B cells that also can be targeted by KIO-100 (PP-001) in uveitis is not yet clear, it varies from promoting T cell activity and its function as antigen-presenting cells to regulation by their production of IL-10 and IL-35 (41–44). In rare cases of therapy-refractive juvenile uveitis or uveitis accompanying multiple sclerosis targeting B cells has shown some effect (45–48). Nevertheless, since inhibition of DHODH also impedes B cell activation, the therapeutic effect of KIO-100 (PP-001) exceeds an exclusive T cell suppression as gained by the presently approved therapies for uveitis, ciclosporin A and TNF-blocker Adalimumab (2).

The effect of intraocular KIO-100 (PP-001) is primarily the suppression of T cell activation, which is in coincidence with previous *in vitro* data of peripheral blood lymphocytes from human donors showing that KIO-100 (PP-001) suppresses proliferation of lymphocytes and their cytokine secretion, but has no effect on the secretion of monokines, suggesting that monocytes/macrophages are not affected by DHODH inhibition (15).

The number of patients in this first clinical trial of KIO-100 (PP-001) in humans was limited by design, and therefore too small to attribute certain positive effects to a clinical diagnosis or disease activity. To answer those questions further studies aiming at clinical efficacy are required to fully explore the potential of the small molecule DHODH inhibitor in the treatment of inflammatory eye disease and cystoid macular edema.

## Data availability statement

The original contributions presented in this study are included in the article/Supplementary material, further inquiries can be directed to the corresponding author.

## Ethics statement

The studies involving human participants were reviewed and approved by the Ethical Committee of the LMU Münich, Pettenkoferstr. 8a, 80336 München, Germany (CEC). The patients/participants provided their written informed consent to participate in this study.

## Author contributions

ST, FO, RS-Z, SS, and GW had drafted the study protocol. ST, CD, AH, UP, JV, and TB-A had acquired and documented the patient’s data. ST, FO, SS, RS-Z, and GW had conducted the preclinical studies. ST and GW had resumed the clinical study data and written the manuscript. ST had designed the figures. All authors have read and revised the manuscript and approved the submitted version.
