# Supplementary material for: A new small molecule DHODH-inhibitor [KIO-100 (PP-001)] targeting activated T cells for intraocular treatment of uveitis — A phase I clinical trial
Source: Front Med (Lausanne). 2022 Oct 17;9:1023224. doi: 10.3389/fmed.2022.1023224 (PMC9621317; doi:10.3389/fmed.2022.1023224)
Supplement: Supplementary file 1 [file Data_Sheet_1.PDF]

A new small molecule DHODH-inhibitor (KIO-100 (PP-001)) targeting activated T cells for intraocular treatment of uveitis — a phase I clinical trial

SUPPLEMENTAL DATA

Table S1: List of Ethics Committees of participating sites

UZ Leuven Campus Gasthuisberg Ethische commissie onderzoek, Herestraat 49, 3000 Leuven, Belgium.

Ethik-Kommission der LMU München, Pettenkoferstr. 8a, 80336 München, Germany, (CEC).

Landesamt für Gesundheit und Soziales Berlin Ethik-Kommission, Fehrbelliner Platz, 10707 Berlin, Germany, (LEC).

Ethik-Kommission an der Medizinischen Fakultät der Eberhard-Karls-Universität und am Universitätsklinikum Tübingen, Gartenstraße 47, 72074 Tübingen (LEC), Germany.

Ethik-Kommission Medizinische Universität Wien, Broschkegasse 8b/6, 1090 Wien, Austria.

Ethik-Kommission der Ärztekammer Westfalen-Lippe und der Westfälischen Wilhelms-Universität Münster, Gartenstraße 210 – 214, 48147 Münster, Germany (LEC).

Table S2: List of inclusion and exclusion criteria

Inclusion Criteria

- Male or female patients 18-64 years of age who have diagnosis of chronic posterior uveitis, intermediate uveitis or panuveitis.
- Good general state of health (mentally and physically).
- A signed and dated written informed consent form.
- A signed and dated written data protection consent form.
- Female patients of childbearing potential must perform a negative urine pregnancy test within 7 days prior to the injection visit (Day 0).
- Male and female patients must ensure that two acceptable methods of contraception are used for the entire duration of the study, from first dose up to the study follow-up visit, and refrain from becoming pregnant or fathering a child in the 3 months following the last study drug administration. Male patient must agree with their female partners prior to screening to use a medically accepted method of contraception while receiving protocol-specified medication, and for 3months after stopping the medication. Highly effective methods of birth control are defined as those that result in a low failure (i.e., < 1%per year) when used consistently and correctly, such as hormonal implants, injectable contraceptives, combined oral contraceptives, hormonal intrauterine devices, or surgical sterilization. One of the contraceptive methods must be a barrier method e.g., condom. Periodic abstinence and withdrawal are not acceptable methods of contraception. Female partners of male patients must be informed by their male partners about the need to use highly effective methods of birth control as defined above.
- Have diagnosis of chronic posterior uveitis, intermediate uveitis or panuveitis (as defined by the Standardization of Uveitis Nomenclature Working Group (18) in at least one eye. For patients with panuveitis, the anterior component of inflammation must be less than the posterior component. The investigator to his best knowledge must rule out any suspected masquerade syndrome or infection prior to study entry.
- Have chronic, posterior uveitis, intermediate uveitis or panuveitis with uncontrolled inflammation.
- Have media clarity, pupillary dilation, and patient cooperation sufficient for adequate visualization of the optic nerve in the study eye.
- Have been receiving a stable dose therapy of systemic corticosteroid treatment or immunosuppressive therapy (azathioprine, methotrexate, cyclosporine, mycophenolate, tacrolimus) or any combination thereof for at least 3 months prior to study inclusion.
- Best-corrected Early Treatment Diabetic Retinopathy Study (ETDRS) visual acuity of 10 letters or better (approximately 1/35 or 0.032) but less than 50 letters (approximately 20/100 or 0.2) in the study eye.
- Best-corrected ETDRS visual acuity of 70 letters or better in the fellow eye (approximately 20/40 or 0.5).

Exclusion Criteria

- Patients in whom media opacities (cornea, anterior or posterior synechia, cataract, vitreous haze, and others) of either eye preclude investigation and documentation of the posterior pole and intravenous fluorescein angiography, or optical coherence tomography evaluation in the study eye.
- Patients receiving any local or systemic biologicals (i.e., tumor necrosis factor [TNF]- blockers, B cell-blockers, cytokines, cytokine-blockers, receptor antagonists).
- Treatment with cyclophosphamide or chlorambucil.
- Intravitreal injections (including but not limited to anti-vascular endothelial growth factors) 60 days prior to the baseline.
- Posterior subtenon's injection or orbital floor injection of steroids 90 days prior to baseline. Any implantable corticosteroid-eluting device (Ozurdex, Iluvien, Retisert, triamcinolone intravitreal implant, fluocinolone intravitreal implant) in the study eye, with the following exceptions:
    - If the device had been removed more than 90 days prior to Day 0 of this study, the eye will be eligible for PP-001-1001.
    - If Ozurdex had been implanted 6 months before Day 0 of this study, the eye will be eligible for PP-001-1001.
    - If Iluvien or Retisert had been implanted 3 years before Day 0 of this study, the eye will be eligible for PP-001-1001.
- Intraocular surgery within 90 days prior to Day 0 in the study eye.
- Capsulotomy within 30 days prior to Day 0 in the study eye.
- History of vitreoretinal surgery or scleral buckling within 90 days prior to Day 0 in the study eye.
- Any ocular surgery (including cataract extraction or capsulotomy) of the study eye anticipated within the first 60 days following Day 0.
- Intraocular pressure (IOP) >25 mmHg in the study eye (glaucoma patients maintained on no more than one topical medication with IOP <25 mmHg are allowed to participate).
- Ocular hypotony (IOP less than 6mmHg).
- Pupillary dilation inadequate for quality fundus photography in the study eye.
- Aphakia or anterior chamber lens in the study eye (posterior chamber lens was acceptable).
- Visible scleral thinning, scleral ectasia, or keratoconus in the study eye.
- Presence of any ocular malignancy.
- Ocular or periocular infection in either eye or the use of systemic antibiotics.
- Participation in other investigational drug or device clinical trials within 90 days prior to Day 0 or planning to participate in other investigational drug or device clinical trials within 180 days following Day 0. This includes both ocular and non-ocular clinical trials.
- Female patients who are pregnant, nursing, or planning a pregnancy, or who are of childbearing potential and not willing to use reliable means of contraception.
- Use of any anticoagulant or thrombocyte aggregation inhibiting agent (marcumar, warfarin, heparin, enoxaparin, apixaban, rivaroxaban, pentosan polysulfate, dabigatran, aspirin, and others) less than 14days prior to injection visit (Day 0).

- Known allergy or hypersensitivity to the study medication, any component of the delivery vehicle, any corticosteroids or any diagnostic agents used during the study (e.g., fluorescein, dilation drops, antibiotic drops, povidone).

Legends to supplemental Figures:

Fig. S1: Intraocular pressure (IOP) from baseline until the end of the study at day 28, assessed at the respective visits. Mean mmHg ± SE is shown for all patients in each treatment group (n=4).

Fig. S2: Inflammatory cells in the anterior chamber (AC) from baseline until the end of the study at day 28, assessed at the respective visits. Mean grading score of cells ± SE is shown for each visit and each group as designated. According to SUN grading scheme for AC cells 6 grading steps are defined (0+, 0.5+, 1+, 2+, 3+, and 4+).

Fig. S3: Vitreous haze, according to SUN grading from baseline until the end of the study at day 28, assessed at the respective visits. Mean scores ± SE are shown for each visit and each group as designated. Vitreal haze grading according to SUN consists of 6 ordinal grading steps (0+, 0.5+, 1+, 2+, 3+, and 4+) (1, 2).

Fig. S4: Central retinal thickness (CRT) from baseline until the end of the study at day 28, assessed at the respective visits. CRT measurements were extracted from automated standard OCT (optical coherence tomography) of the central fovea of the macula. Two patients in the group receiving 1.2 µg KIO-100 (PP-001) had a macular atrophy at inclusion and therefore the OCT value in this group were subnormal (normal central retinal thickness is 250 to 270 µm).

Fig. S5: Visual field testing was performed at baseline, days 7 and 28. All patients had loss of visual field sensitivity at baseline due to the destruction of previous chronic uveitis. In the follow up the were no additional losses of more the 2 dB in single patients. On day 28 the average visual field sensitivity had increases slightly, but this is partially due to training effects.

Fig. S6: Corneal endothelial cells counts per mm^2. In none of the 3 dosing groups a trend towards cell loss was detected.
