# Supplementary figures and images for: A new small molecule DHODH-inhibitor [KIO-100 (PP-001)] targeting activated T cells for intraocular treatment of uveitis — A phase I clinical trial
Source: Front Med (Lausanne). 2022 Oct 17;9:1023224. doi: 10.3389/fmed.2022.1023224 (PMC9621317; doi:10.3389/fmed.2022.1023224)

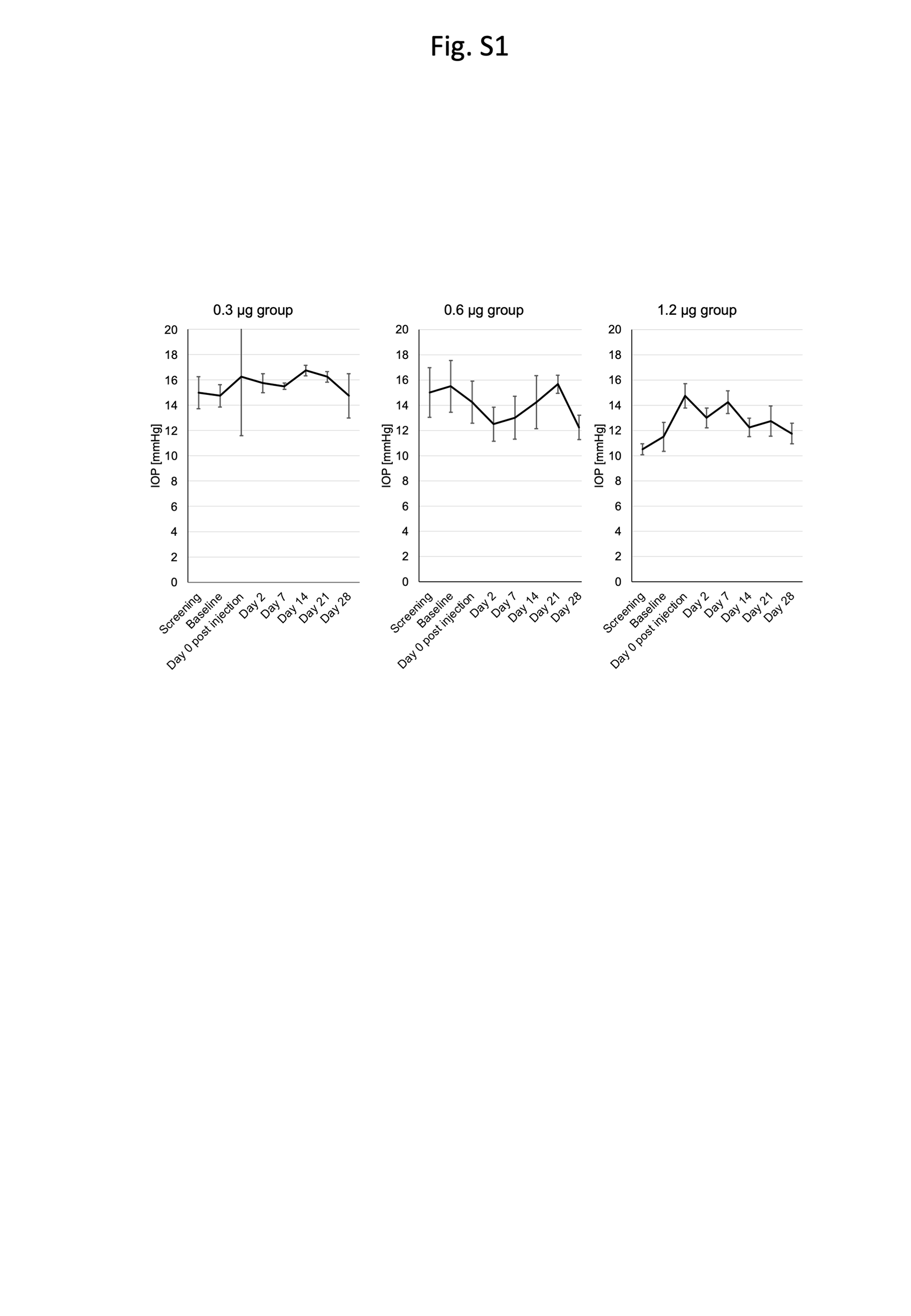

Supplement: Supplementary file 2 [file Image_1.TIF]

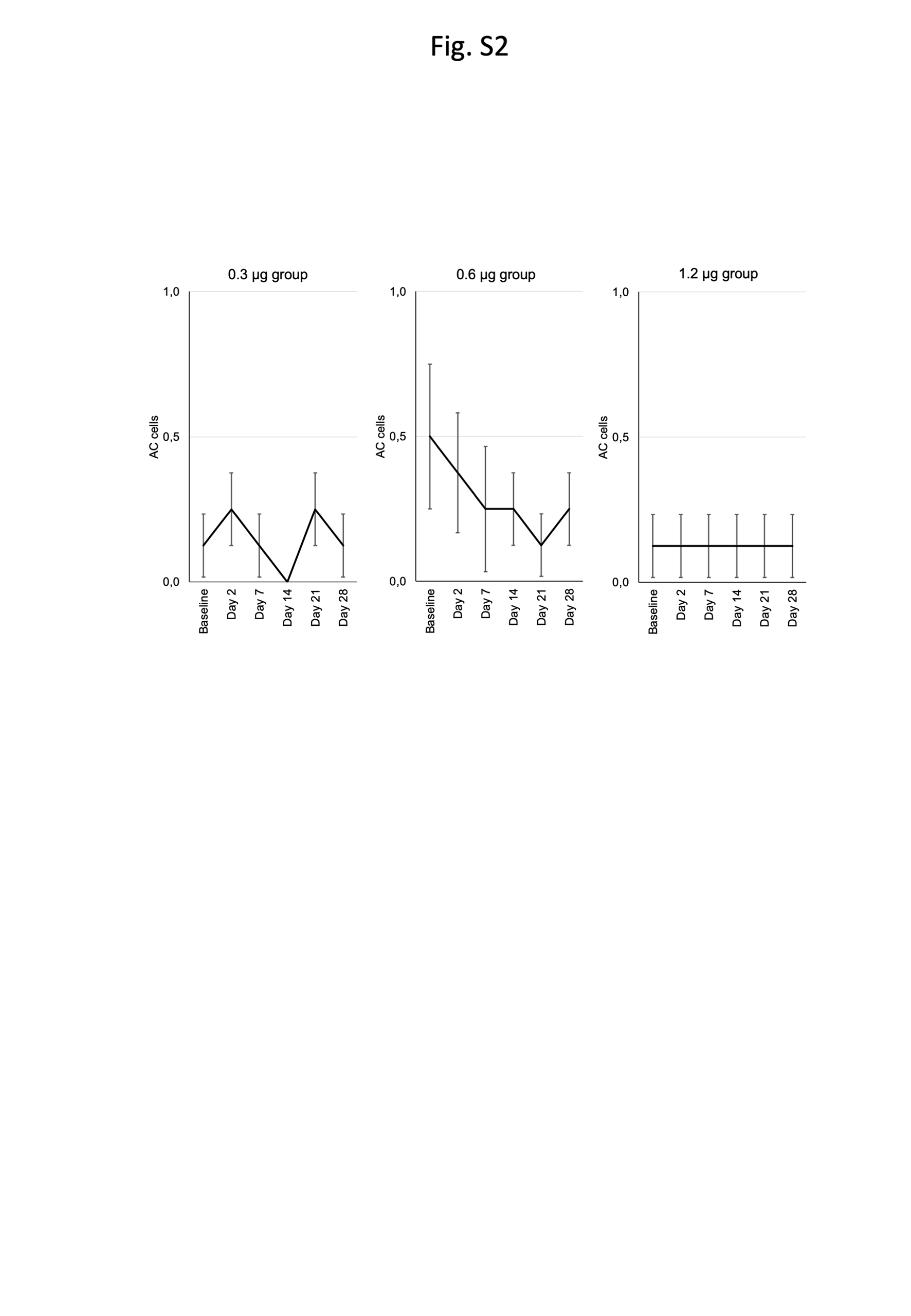

Supplement: Supplementary file 3 [file Image_2.TIF]

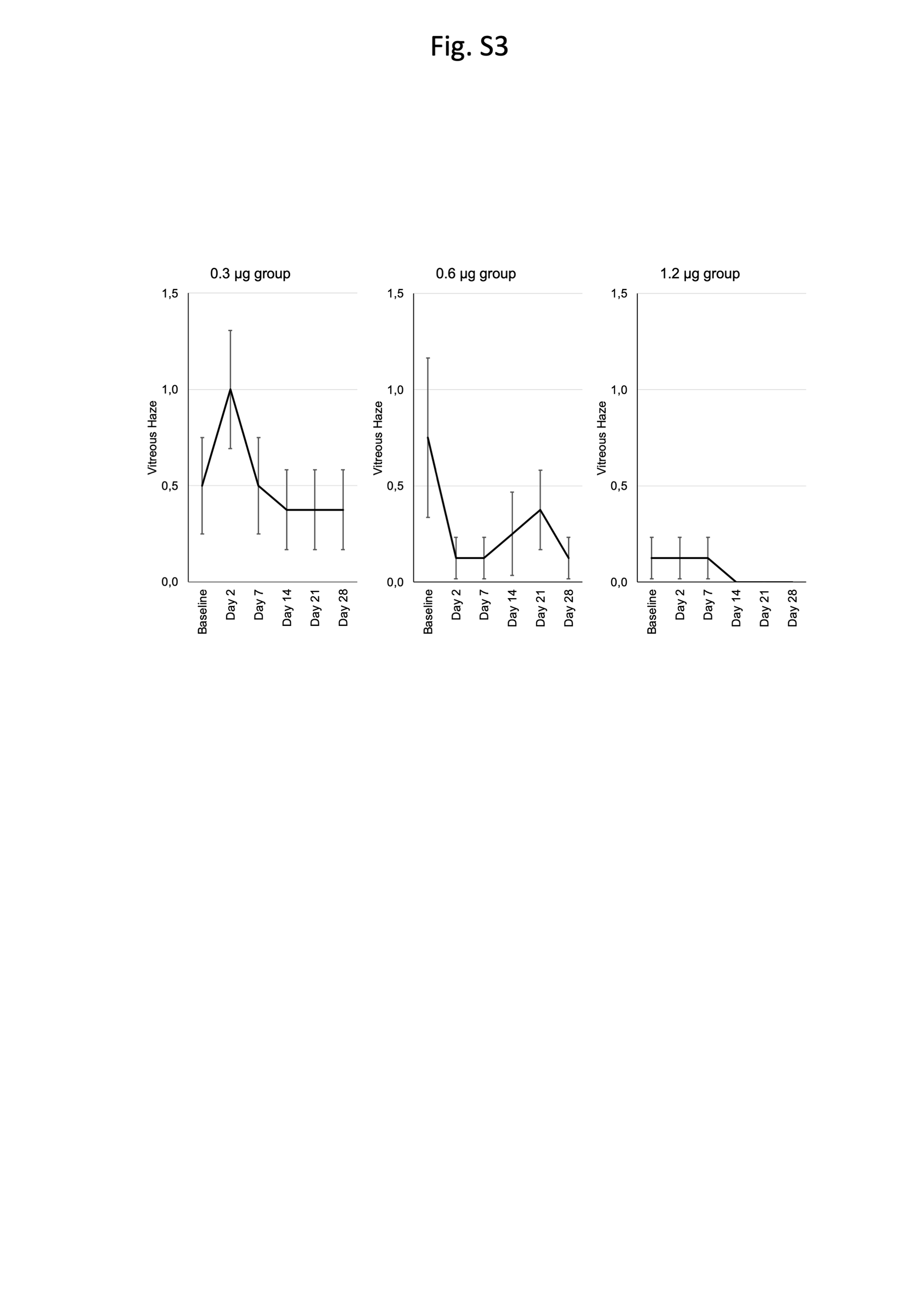

Supplement: Supplementary file 4 [file Image_3.TIF]

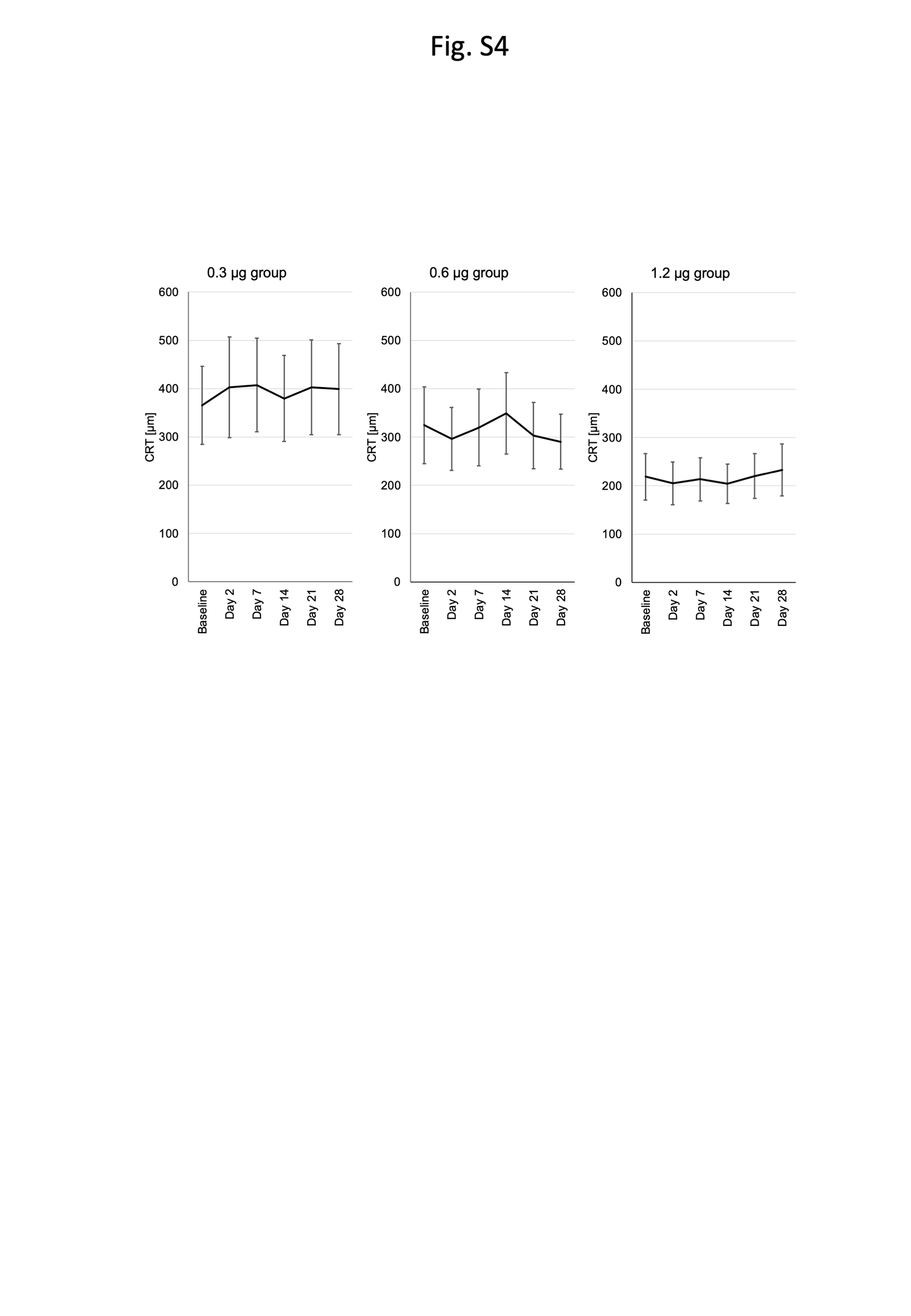

Supplement: Supplementary file 5 [file Image_4.TIF]

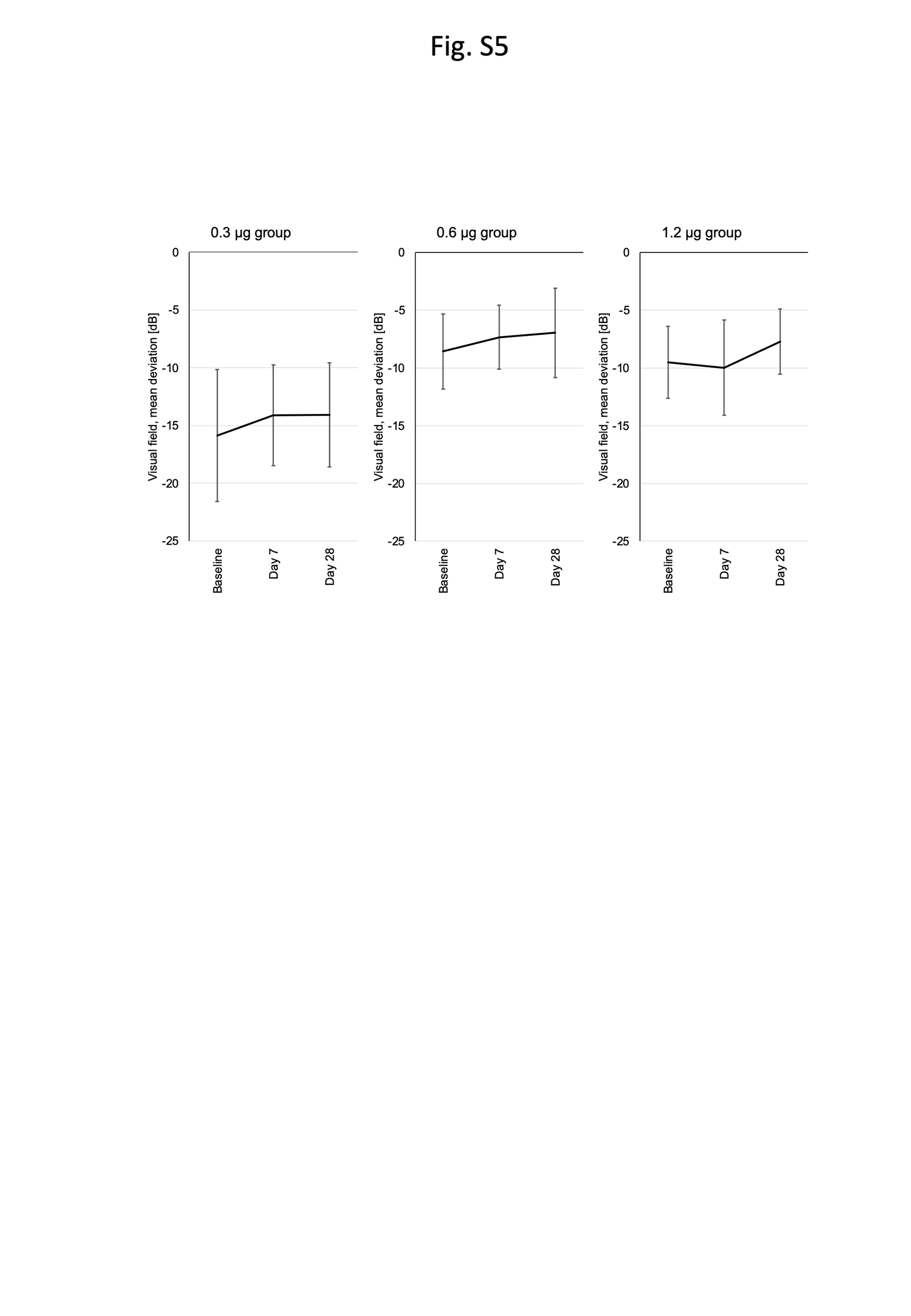

Supplement: Supplementary file 6 [file Image_5.TIF]

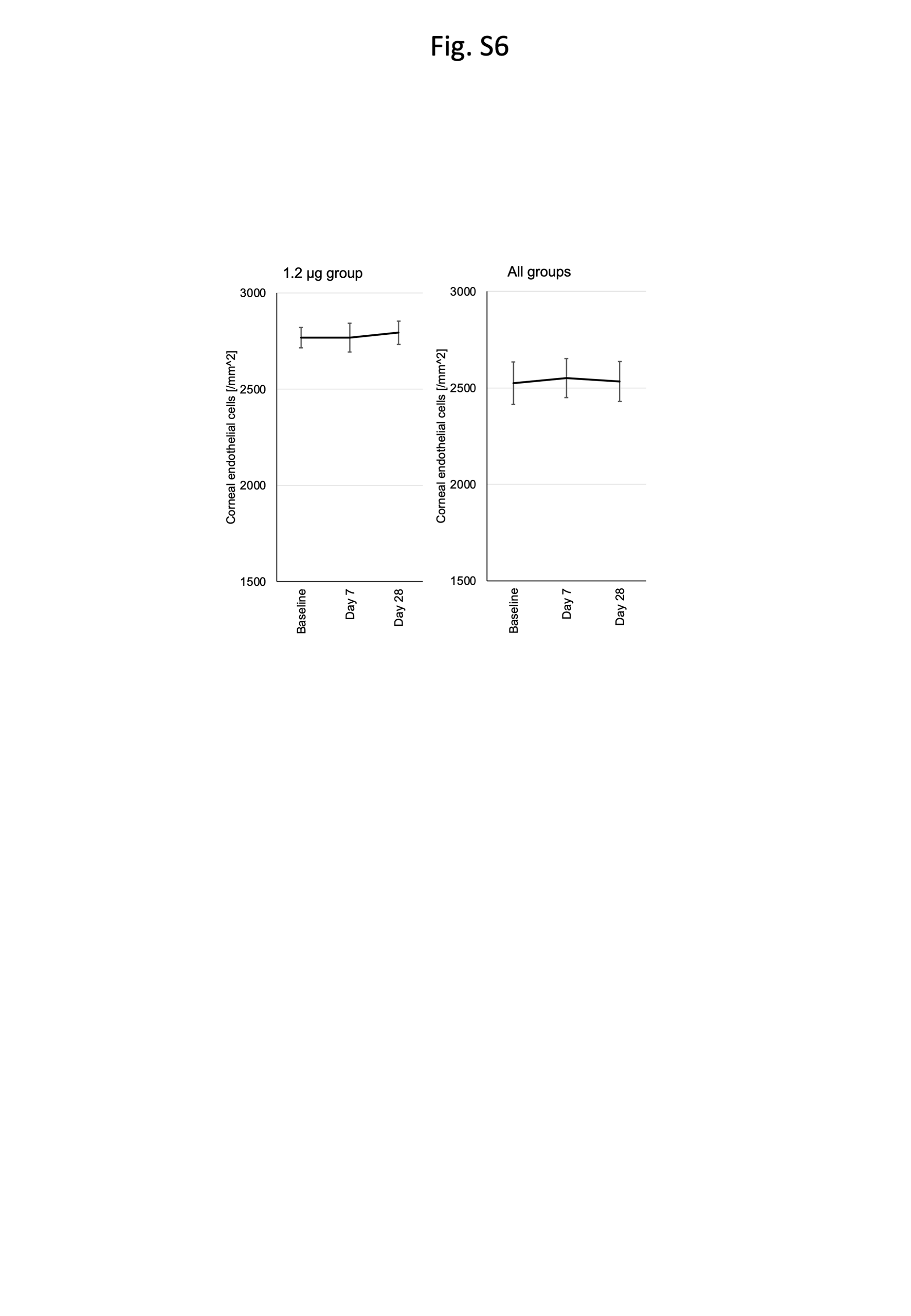

Supplement: Supplementary file 7 [file Image_6.TIF]
